# Supporting Virtual Dermatology Consultation in the Setting of COVID-19

**DOI:** 10.1007/s10278-021-00425-6

**Published:** 2021-03-10

**Authors:** Allison Kutner, Danielle Love, Alina Markova, Anthony Rossi, Erica Lee, Kishwer Nehal, Mario Lacouture, Veronica Rotemberg

**Affiliations:** Memorial Sloan Kettering Cancer Center, 1250 York Avenue, 10065 New York, NY USA

**Keywords:** Telemedicine, Teledermatology, COVID-19, DICOM

## Abstract

While telemedicine has been utilized with more frequency over the past two decades, there remained significant barriers to its broad implementation. The COVID-19 global pandemic served as a stimulus for rapid expansion and implementation of telemedicine services across medical institutions worldwide in order to maximize patient care delivery, minimize exposure risk among healthcare providers and patients alike, and avoid overcrowding of patient care facilities. In this experience report, we highlight the teledermatology initiatives executed by the Dermatology Service at Memorial Sloan Kettering Cancer Center in New York City, with particular emphasis on image ingestion and potential for future automation and improvement.

## Background

Virtual physician-patient visits provide significant opportunity to advance delivery of care in a visual specialty such as dermatology, however, prior to March 2020, were significantly underutilized [1]. In particular, telemedicine has proven to be a reliable consultation tool [2], increases access to underserved populations [3], improves efficiency [3], and is cost effective [4]. In some reports, up to 88% of patients are highly satisfied with teledermatology encounters with their provider [5–7]. Telemedicine has specifically high potential in public health emergencies, and to date, it has been underutilized [8]. Despite the trend toward increased utilization of telemedicine, progress within many healthcare systems has been slow, as there have historically been many barriers to widespread telemedicine use [9], including: regulatory burdens, reimbursement limitations, and paucity of reliable technology platforms.

The Dermatology Service at Memorial Sloan Kettering Cancer Center (MSK) provides dermatology services for patients undergoing oncologic care [10] and manages patients diagnosed with and at high risk for cutaneous malignancies. Given the exigencies of these types of dermatologic patient needs [11], patient care, in both person and virtual, has dramatically changed over the past few months. Caring for oncology patients’ dermatologic needs and those with cutaneous malignancies remotely became a priority over a very rapid period in March 2020, as New York City (NYC) became a “hot spot” for COVID-19 cases [12]. Priorities such as conservation of personal protective equipment (PPE) and concern for healthcare providers and healthcare resources meant that the majority of patients scheduled to be seen in outpatient clinical settings needed to be seen, or at least triaged, remotely.

We outline how, with the support of administration, clinicians, information technology, and nursing, we were able to rapidly implement teledermatology services for patients at multiple locations in order to continue providing critical patient care. The expansion of reimbursement for telemedicine by the Centers for Medicare and Medicaid Services (CMS), while beyond the scope of this work, also supported our efforts to implement store-and-forward static image-based consultations, live face-to-face teledermatology, and telephone encounters.

**Fig. 1:**
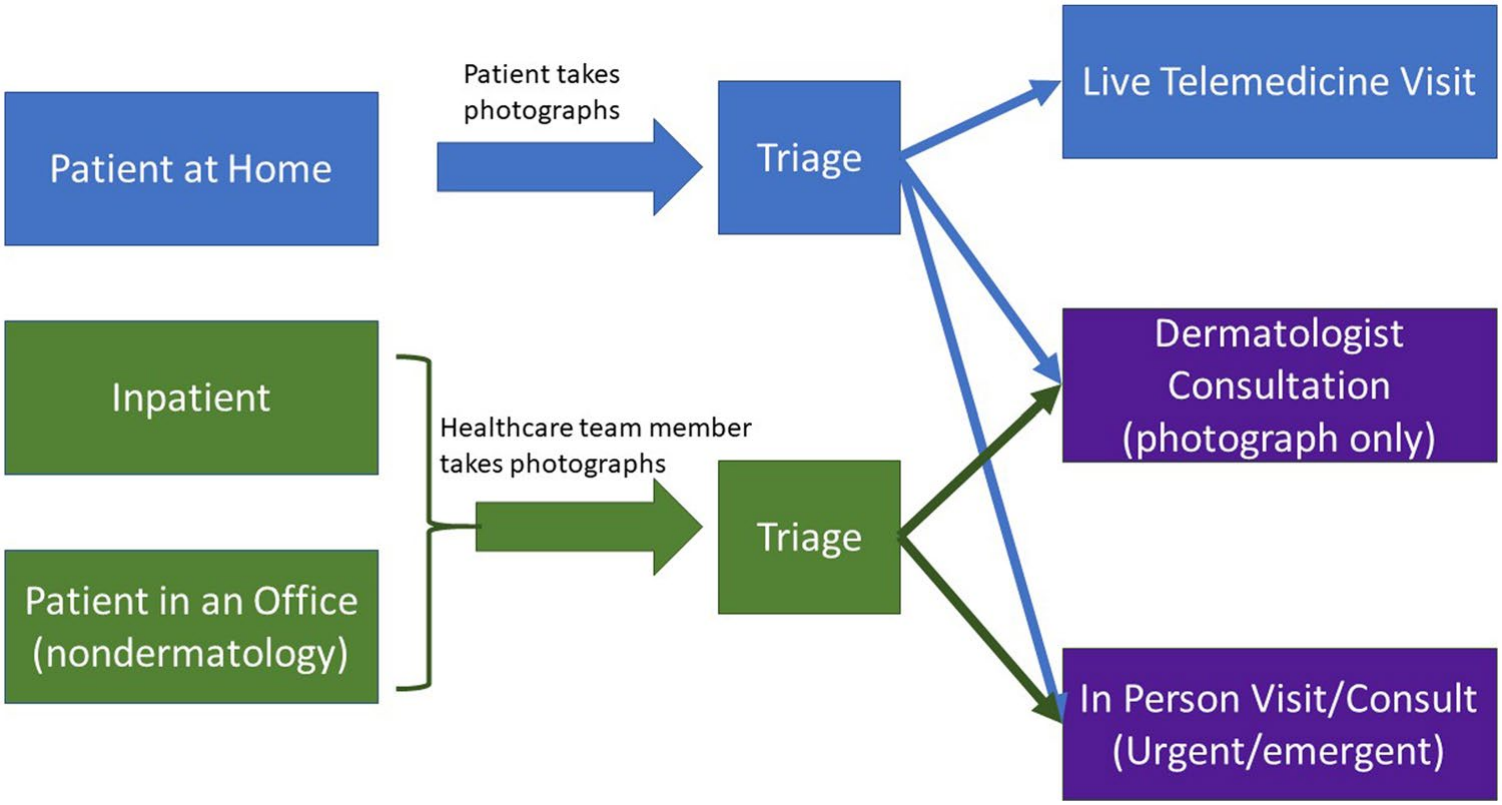
The left side of the diagram highlights the three possible patient scenarios we envisioned: patient at home, inpatient (patients admitted to the hospital), and patients in a non-dermatologist’s office. In order for a patient to have dermatologic consult implemented either virtually or in person, photographs were requested, and triage was performed as shown

We addressed three patient scenarios: patient at home, patient in an office that was not a dermatology office, and patient admitted to the hospital (inpatient), for which teledermatology services were desirable. We also implemented three types of dermatology responses: live telemedicine visit, store-and-forward dermatology consultation, and a traditional in-person visit for urgent/emergent cases to these scenarios, which are highlighted in Fig. 1.

In this report, we describe the implementation for teledermatology services by the Dermatology Service at Memorial Sloan Kettering Cancer Center (MSK) in response to the COVID-19 global pandemic.

## Methods

We evaluated the expansion of teledermatology from March 13, 2020, until June 13, 2020. These dates were selected as schools closed in New York Statewide on March 16, a closure that reflected a significant decrease in the in-person volume in our dermatology clinic, and as New York City began phase I reopening on June 8, after which clinic volume has gradually begun to normalize.

Imaging: In order to triage the patients that would be appropriate for different virtual or in person visit types, photographs were requested from patients requiring dermatologic evaluation as shown in Fig. 1. Patients were imaged photographically primarily using smartphone cameras.

Several patient education resources were created for at home imaging while internal resources with instructions were also generated for healthcare team members requesting dermatologic consultation [13–15].

Patients at home uploaded their photos to a secure patient portal, while healthcare team members were encouraged to send photos to the appropriate (outpatient or inpatient) on-call dermatology consult team created for this purpose in a Health Insurance Portability and Accountability (HIPAA)-compliant Voalte™ (Voalte, Sarasota, FL) application.

Once photos were received, administrative and nursing team members fielded the messages, and photographs with clinical history were reviewed by the physician via email or in the Voalte™ system directly in order to determine the appropriate visit type.

Triage: Dermatologists at MSK based their decision on the type of visit to perform primarily based on images supplied by patients or referring providers, as well as clinical information. Due to the rapidity with which teledermatology was implemented, triage was primarily performed by physicians directly; however, ongoing efforts are being put into place to improve efficiency and automation of patient triage based on types of dermatologic requests. For example, our dermatologic surgeons are frequently performing initial consultations for known cutaneous malignancies via telemedicine visits to triage low-risk and high-risk lesions and determine the appropriate timing of surgical treatment. Store-and-forward images were reviewed in advance to assess for site identification and lesion assessment. Post-surgical follow-up and scar images were further triaged by nursing, and physicians would then determine which patients would benefit from a live telemedicine call.

Image Ingestion: All images reviewed were ultimately uploaded into our secure image database, Vectra™ (Canfield Scientific, Parsippany, NY) imaging software, by a member of the office or clinical staff. For patients at home, portal messages were directly retrieved, saved to an intermediate on-campus secure desktop, and uploaded to the imaging database. The lack of imaging standards for dermatology to guide interoperability, metadata, color, and scale, required upload to proprietary software be performed manually for this initial deployment [16]; however, future iterations could become more automated and release photos in a wider distribution. The Voalte™ desktop application was used daily to retrieve and upload photographs directly by the office staff assigned to the specific dermatology consult team.

Teledermatology Consultation: Dermatologists provided both synchronous (real-time) and asynchronous (time-disparate, store-and-forward) patient-provider interactions and provider-to-provider virtual consultations. Due to the expansion of potential telemedicine platforms and relaxation of HIPAA protections by CMS, clinicians were able to use Cisco Jabber (Cisco Systems, San Jose, CA), Doximity, and a variety of other face-to-face methods to communicate with patients (i.e., Apple FaceTime). We also enabled direct telephone consultation for patients who sent in photographs via portal message.

## Results

**Fig. 2:**
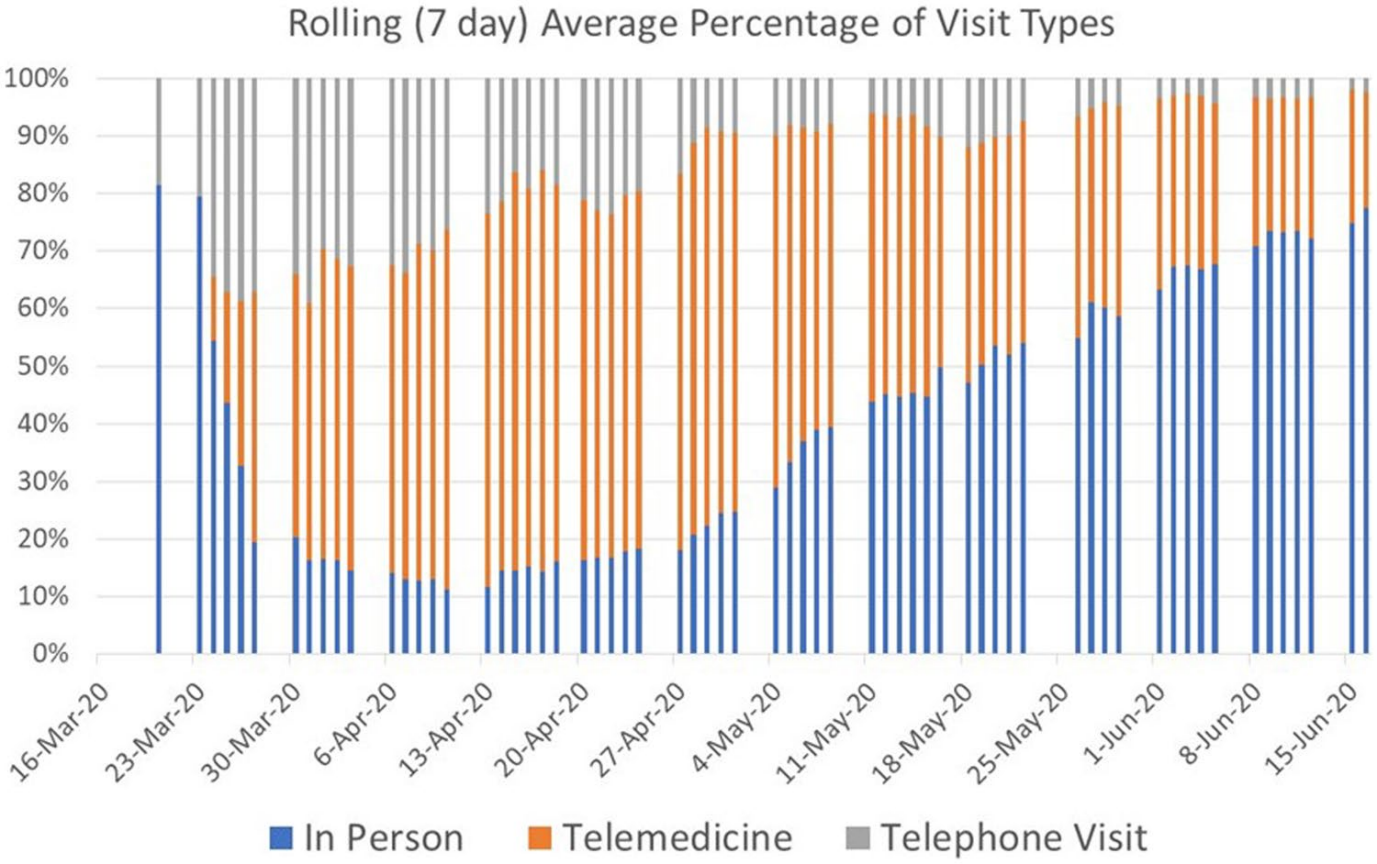
Proportion of total visits conducted (7-day average) that were performed via telemedicine (face to face video) or telephone visits (supplemented by static photographs). As the number of positive SARS-CoV2 tests in NYC began to decrease, in person visits also began to increase at our center. However, in June, we continued to have 20–30% of our visits performed virtually, which may represent a new steady state

Figure 2 shows the percentage of total dermatology outpatient clinic visits that were able to be converted to telemedicine or telephone visits in April and May of 2020. In early March, none of the visits to the dermatology service were telemedicine visits, whereas in June, as clinical in-person volume increased, telemedicine visits reflected 20% of total clinical volume. This 20% could reflect an ongoing enthusiasm for telemedicine visits or continued limitations on in-person clinic volume.

**Fig. 3:**
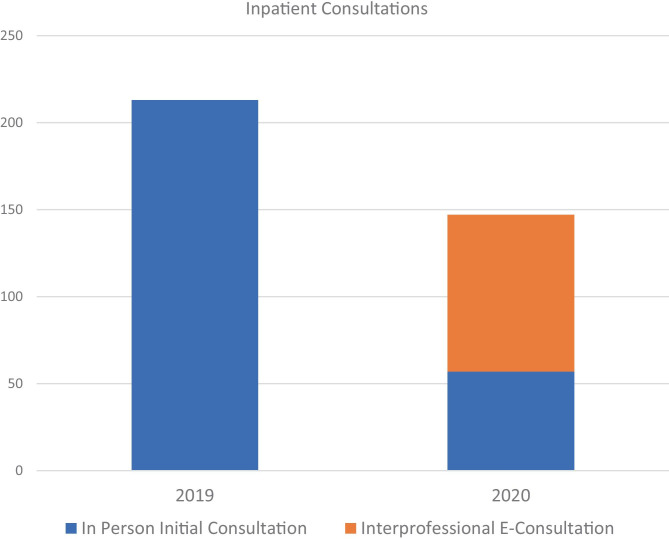
Over the 3-month period of observation between March 13, 2020, and June 13, 2020, 61% of initial inpatient consultations were performed via interprofessional e-consultation in 2020 as compared with zero in 2019

Figure 3 shows the proportion of inpatient consultations, where PPE was at a particular premium, that were able to be converted to interprofessional e-consults on the basis of smartphone photography and secure messaging on the Voalte™ platform. Over the 3-month period, 61% of inpatients requiring dermatology evaluation were seen via remote store and forward e-consultation rather than in person.

**Fig. 4:**
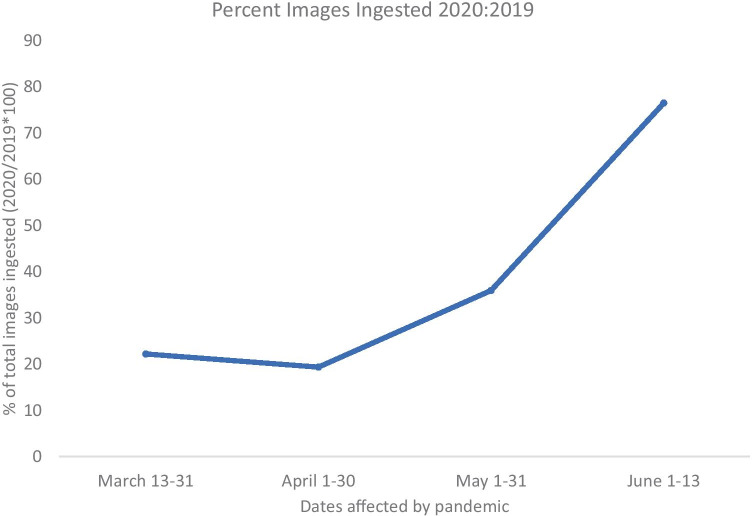
Percentage of photographs ingested to the imaging database in 2020 as compared with 2019 between March 13th and June 13th

Figure 4 shows the proportion of images ingested into the Vectra™ imaging database in 2020 as compared with 2019. This demonstrates that large volumes of images (greater than 10,000) were able to be ingested despite a manual process. The larger image volumes in May and June may partially represent resumption of clinical activities in addition to stabilization of teledermatology visit volume.

## Discussion

Initiation of workflows that relied on secure messaging and patient portal upload allowed dermatologists at MSK provide care to patients while minimizing in-person patient volume to address the impact of COVID-19.

We found that triage of patients via asynchronous (store-and-forward) images provided by patients or healthcare team members as shown in Fig. 1 was particularly critical to our ability to care for oncologic patients during a pandemic in which in-person visits were to be avoided when possible. This is consistent with prior literature on “forward triage,” which refers to the sorting of patients prior to their arrival in the emergency department as is a method of healthcare surge control [17]. This practice was able to substantially decrease unnecessary patient visits and promote social distancing, as has been reported in other settings [18]. This image receipt and storage was critical to decision-making about who should be seen in-person but was performed inefficiently due to the manual upload and rapidity with which virtual dermatology was implemented. The three key areas for automation potential in order to increase enthusiasm and adoption for teledermatology visits would be image capture, ingestion and patient triage.

Over the course of the 3-month observation period, dermatologists at MSK received more than 10,000 photographs acquired by patients or their families via smartphone applications or other mobile devices. The image quality, despite patient education materials, was highly variable. Further development of image standards such as Digital Imaging and Communications in Medicine (DICOM) for dermatology imaging could also improve interoperability and security of image ingestion [16]. An automated capture or ingestion pipeline with real-time image quality feedback along with a ruler or cuboid for measurement would greatly increase the diagnostic potential of these patient-captured images.

Dermatologists at MSK reviewed images through secure messaging, email, and portal messages that were uploaded manually to our image viewing software. Future automation efforts could be devoted to triage approaches and image ingestion. Clinicians observed that they were frequently reviewing patient cases twice: first during triage and subsequently during a televisit. Automated approaches to quantify body surface area affected by skin disease, flag abnormal vital signs, and visit types or patient questions could improve the efficiency and perhaps largely automate the process of patient triage.

One can envision a scenario in which images are automatically ingested to the image database through those three streams of receipt and matched to patient identification and anatomic site. At that point, clinicians could simply review images in one master database rather than monitoring multiple streams of communication, and physician observations would be documented in the electronic medical record (EMR). This could apply for store-and-forward teledermatology visits as well with the option to document directly in the image storage platform that seamlessly links the images and the EMR. As shown in Fig. 3, we found teledermatology in the inpatient setting, especially store-and-forward interprofessional e-consultations (IPECs) to be utilized at a high volume. These types of consultations enabled referring providers to minimize exposure risks to themselves, colleagues and patients, and permitted a collective attempt to minimize unnecessary PPE use. The inpatient teams are also familiar with using the HIPAA-compliant Voalte™ messaging service for other purposes, which may have contributed to a high rate of adoption and reduced the clinician effort since triage and consultation were performed simultaneously for virtual visits.

At the end of the three-month observation period, outpatient visits were still being performed via virtual consultation 20–30% of the time, which likely reflects ongoing enthusiasm for teledermatology as well as a lack of complete return to clinic volume. Further efforts toward automation, image standards, and efficiency will support continued development and response to potential future crises.

## Conclusions

We successfully implemented teledermatology workflows that provided continued access to dermatologic care during the COVID-19 global pandemic. We achieved this using a variety of methods (both store-and-forward teledermatology and live telemedicine platforms), in various settings (inpatient and outpatient), as well as with different key players (patient-to-provider, and provider-to-provider). Forward triage telemedicine offers a patient-centered approach that in a public health crisis such as SARS-CoV-2 protects patients, healthcare providers, and the community while supporting uninterrupted patient care [17]. Despite barriers related to image quality and storage, inadequate technological platforms to conduct patient visits, and confusing workflows, we swiftly implemented several telemedicine platforms to increase our reach to patients paying specific attention to the visual aspect of dermatology and its unique need for precise imaging. It is important for key agencies such as CMS and the US Department of Health and Human Services (HHS) to embrace utilization and implementation of telemedicine going forward.
